# Allergic rhinitis

**DOI:** 10.1186/s13223-024-00923-6

**Published:** 2024-12-27

**Authors:** Lana Rosenfield, Paul K. Keith, Jaclyn Quirt, Peter Small, Anne K. Ellis

**Affiliations:** 1 Department of Internal Medicine, University of Manitoba, Winnipeg, MB Canada; 2 Division of Clinical Immunology and Allergy, Department of Medicine, McMaster University, Hamilton, ON Canada; 3 Jewish General Hospital, Montreal, QC Canada; 4 Division of Allergy & Immunology, Department of Medicine, Queen’s University, Kingston, ON Canada

## Abstract

Allergic rhinitis (AR) is a common disorder that is strongly linked to asthma and conjunctivitis. Classic symptoms include nasal congestion, nasal itch, rhinorrhea and sneezing. A thorough history, physical examination and assessment of allergen sensitization are important for establishing the diagnosis of AR. Intranasal corticosteroids and second-generation antihistamines are the mainstay of treatment. Allergen immunotherapy is an effective immune-modulating treatment for use in addition to or as an alternative to pharmacologic therapy. This article provides an overview on the pathophysiology, diagnosis, and appropriate management of AR.

## Key take-home messages

- AR is strongly linked with asthma and conjunctivitis.
- Allergen skin testing is the best diagnostic test to confirm AR. However, serum-specific IgE testing is a reasonable alternative.
- Intranasal corticosteroids are the mainstay of treatment for most patients that present to physicians with AR. Second-generation oral antihistamines may also be used as first-line therapies.
- Allergen immunotherapy is an effective immune-modulating treatment in conjunction with or as an alternative to pharmacologic therapy.
- At any point in the management of patients with AR, referral to allergist should be considered for confirmatory diagnosis and treatment/management discussions.

## Introduction

Rhinitis is broadly defined as inflammation of the nasal mucosa. It is a common disorder that affects up to 40% of the population [1]. Allergic rhinitis (AR) is the most common type of chronic rhinitis, affecting up to 20% of the Canadian population [2, 3], and evidence suggests that the prevalence of the disorder is increasing [4]. Severe AR has been associated with significant impairments in quality of life (QoL), sleep and work performance [4].

In the past, AR was considered a disorder localized to the nose and nasal passages, but more recent evidence indicates that it may represent a component of a systemic airway disease involving the entire respiratory tract [5]. There are several physiological, functional and immunological relationships between the upper (nose, nasal cavity, paranasal sinuses, Eustachian tube, pharynx and larynx) and lower (trachea, bronchial tubes, bronchioles and lungs) respiratory tracts. For example, both tracts contain a ciliated epithelium consisting of goblet cells that secrete mucous, which serves to filter the incoming air and protect structures within the airways. Furthermore, the submucosa of both the upper and lower airways includes a collection of blood vessels, mucous glands, supporting cells, nerves and inflammatory cells. Evidence has shown that allergen provocation of the upper airways not only leads to a local inflammatory response, but may also lead to inflammatory processes in the lower airways, and this is supported by the fact that rhinitis and asthma frequently coexist [1, 5, 6]. Therefore, AR and asthma appear to represent a combined airway inflammatory disease, and this needs to be considered to ensure the optimal assessment and management of patients with AR [1, 6].

Comprehensive and widely accepted Canadian guidelines for the diagnosis and treatment of AR were published in 2007 [1]. This article provides an overview and update on the recommendations provided in these guidelines as well as a review of current literature related to the pathophysiology, diagnosis, and appropriate management of AR. Also included are conclusions from the “Focused Allergic Rhinitis Practice Parameter for Canada” published in 2024 [7].

## Pathophysiology

In AR, numerous inflammatory cells, including mast cells, CD4-positive T cells, B cells, macrophages, and eosinophils, infiltrate the nasal lining upon exposure to an inciting allergen (most commonly airborne dust mite particles, cockroach residues, animal dander, moulds, and pollens). In allergic individuals, the T cells infiltrating the nasal mucosa are predominantly T helper 2 (Th2) in nature and release cytokines (e.g., interleukin [IL]-3, IL-4, IL-5, and IL-13) that promote immunoglobulin E (IgE) production by plasma cells. Crosslinking of IgE bound to mast cells by allergens, in turn, triggers the release of mediators, such as histamine and leukotrienes, that are responsible for arteriolar dilation, increased vascular permeability, itching, rhinorrhea, mucous secretion, and smooth muscle contraction in the lung [1, 4]. The mediators and cytokines released during the early phase of an immune response to an inciting allergen trigger a further cellular inflammatory response over the next 4 to 8 h (late-phase inflammatory response) which results in recurrent symptoms (usually nasal congestion) that often persist [1, 8].

## Classification

Rhinitis is classified into one of the following categories according to etiology: IgE-mediated (allergic), autonomic, infectious and idiopathic (unknown). Although the focus of this article is AR, a brief description of the other forms of rhinitis is provided in Table 1.

**Table 1:**
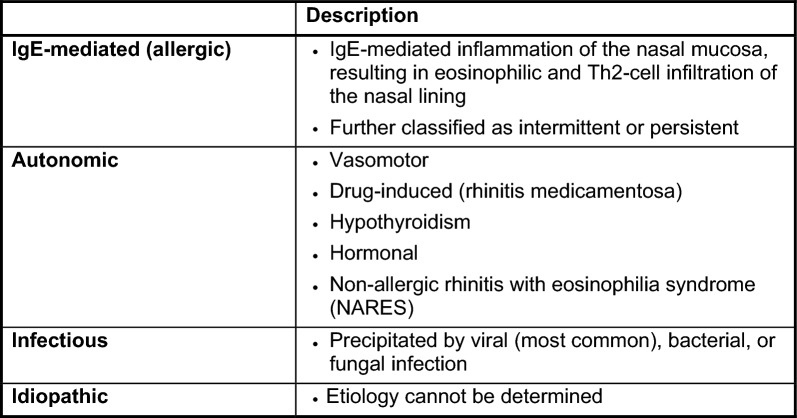
Etiological classification of rhinitis [1]

**Fig. 1:**
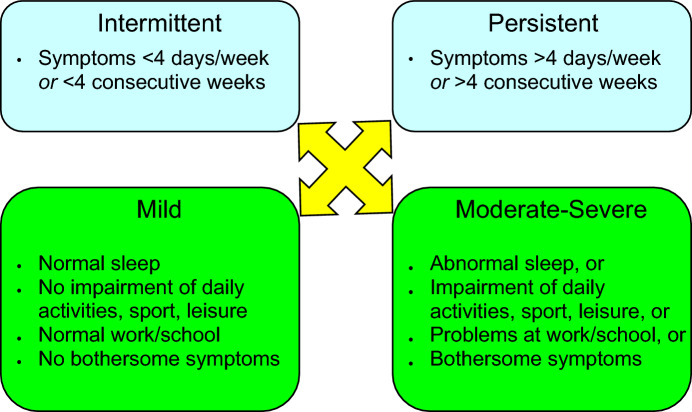
Classification of AR according to symptom duration and severity (Adapted from Small et al. [1], Bousquet et al. [9])

Traditionally, AR has been classified as seasonal (occurs during a specific season) or perennial (occurs throughout the year). However, not all patients fit into this classification scheme. For example, some allergic triggers, such as pollen, may be seasonal in cooler climates, but perennial in warmer climates, and patients with multiple “seasonal” allergies may have symptoms throughout most of the year [8]. Therefore, AR is now classified according to symptom duration (intermittent or persistent) and severity (mild, moderate or severe) (see Fig. 1) [1, 9]. The Allergic Rhinitis and its Impact on Asthma (ARIA) guidelines have defined “intermittent” AR as symptoms that are present less than 4 days per week or for less than 4 consecutive weeks, and “persistent” AR as symptoms that are present more than 4 days/week and for more than 4 consecutive weeks [9]. Symptoms are considered mild when patients have no impairment in sleep and can perform normal activities (including work or school). Symptoms are considered moderate/severe if they significantly affect sleep or activities of daily living, and/or if they are perceived as bothersome. It is important to assess the severity and duration of symptoms as this will guide the management approach for individual patients [1].

Two additional types of rhinitis have been classified that deserve some mention here—occupational rhinitis and local allergic rhinitis.

### Occupational rhinitis

Occupational rhinitis is defined as an inflammatory disease of the nose characterized by intermittent or persistent symptoms that include airflow limitation, hypersecretion, sneezing and pruritus that are attributable to a particular work environment and not to stimuli encountered outside the workplace [10]. Although the overall prevalence of occupational rhinitis is unknown, high-risk professions include laboratory or food-processing workers, veterinarians, farmers and workers in various manufacturing industries [10–12]. Occupational rhinitis usually develops within the first two years of employment. The condition may be IgE-mediated due to allergen sensitization, or due to exposure to respiratory irritants. Symptoms may develop immediately or several hours after exposure to the inciting stimuli. Often there are associated ocular and pulmonary symptoms. An evaluation of the patient suspected of having occupational rhinitis should include the usual history and physical examination (discussed later), as well as a site visit and skin testing or in vitro testing to inhalants. Treatment primarily involves avoiding exposure to the causative agent and pharmacotherapy as needed. There is little evidence to suggest that occupational rhinitis will progress to occupational asthma with ongoing exposure [10, 12], although this is possible. Therefore, patients are generally not advised to leave their jobs if exposure cannot be eliminated but symptoms are adequately controlled.

### Local allergic rhinitis (LAR)

LAR is a clinical entity characterized by a localized allergic response in the nasal mucosa in the absence of evidence of systemic atopy [13–15]. By definition, patients with LAR have a history of symptoms on exposure to perennial and/or seasonal allergens, lack of sensitization found on skin testing or with specific IgE, and a positive nasal allergen provocation test to aeroallergen [16]. It is important to note that the nasal allergen provocation test is not routinely available clinically.

To date, there is no evidence to suggest that LAR is a precursor to AR since follow-up does not show the evolution to typical AR in these patients [17]; however, patient follow-up may not have been long enough to detect this disease evolution. The implications for treatment of LAR are not well understood at this time, although some evidence suggests that allergen immunotherapy may be effective in this type of rhinitis [13, 15].

## Diagnosis and investigations

AR is usually a long-standing condition. Patients suffering from AR often fail to recognize the impact of the disorder on QoL and functioning and, therefore, do not frequently seek medical attention. It is known that patients with perennial symptoms may accommodate and not perceive their symptoms as important [18]. In addition, physicians may overlook regularly questioning patients about the disorder during routine visits [1]. Screening for rhinitis is particularly important in asthmatic patients since studies have shown that rhinitis is present in up to 95% of patients with asthma [19–22].

A thorough history and physical examination are the cornerstones of establishing the diagnosis of AR (see Table 2). Allergy testing is also important for confirming that underlying allergies cause the rhinitis [1]. Referral to an allergist should be considered if the diagnosis of AR is in question.

**Table 2:** Components of a complete history and physical examination for suspected rhinitis [1]

History	Physical examination
*Personal* • Congestion • Nasal itch • Rhinorrhea • Sneezing • Eye involvement • Seasonality • Triggers *Family* • Allergy • Asthma *Environmental* • Pollens • Animals • Flooring/upholstery • Mould • Humidity • Tobacco or cannabis exposure *Medication/drug use* • Beta-blockers • ASA • NSAIDs • ACE inhibitors • Hormone therapy • Recreational cocaine use *Quality of life* • Rhinitis-specific questionnaire *Comorbidities* • Asthma • Mouth breathing • Snoring ± apnea • Impaired smell or taste • Sinus involvement • Otitis media • Nasal polyps • Conjunctivitis *Response to previous interventions* • Avoidance measures • Saline nasal rinses • Second-generation oral antihistamines • Intranasal corticosteroids • Oral or intranasal decongestants • Oral corticosteroids	*Outward signs* • Mouth breathing • Rubbing the nose/transverse nasal crease • Frequent sniffling and/or throat clearing • Allergic shiners (dark circles under eyes) *Nose* • Mucosal swelling, bleeding • Pale, thin secretions • Polyps or other structural abnormalities *Ears* • Generally normal • Pneumatic otoscopy to assess for Eustachian tube dysfunction • Valsalva’s maneuver to assess for fluid behind the ear drum *Sinuses* • Palpation of sinuses for signs of tenderness • Maxillary tooth sensitivity *Posterior oropharynx* • Postnasal drip • Lymphoid hyperplasia (“cobblestoning”) • Tonsillar hypertrophy *Chest and skin* • Atopic disease • Wheezing

*ASA* acetylsalicylic acid, *NSAIDs* non-steroidal anti-inflammatory drugs, *ACE* angiotensin-converting enzyme, *OTC* over-the-counter

### History

During the history, patients will often describe the following classic symptoms of AR: nasal congestion, nasal itch, rhinorrhea and sneezing. Allergic conjunctivitis (inflammation of the membrane covering the white part of the eye) is also frequently associated with AR and symptoms generally include redness, tearing and itching of the eyes [1]. Postnasal drip caused by nasal inflammation may cause cough or a sensation of sputum in the throat.

A detailed history of the patient’s home and work/school environments is recommended to determine potential triggers of AR. The environmental history should focus on common and potentially relevant allergens including pollens, furred animals, textile flooring/upholstery, tobacco or cannabis smoke, humidity levels at home, as well as other potential noxious substances that the patient may be exposed to at work or at home. The use of certain medications [e.g., beta-blockers, acetylsalicylic acid (ASA), non-steroidal anti-inflammatory drugs (NSAIDs), angiotensin-converting enzyme (ACE) inhibitors, and hormone therapy] as well as the recreational use of cocaine can lead to symptoms of rhinitis and, therefore, patients should be asked about current or recent medication and drug use [1].

The history should also include patient questioning regarding a family history of atopic disease, the impact of symptoms on QoL and the presence of comorbidities such as asthma, mouth breathing, snoring, sleep apnea, sinus involvement, otitis media (inflammation of the middle ear), or nasal polyps. Patients may attribute persistent nasal symptoms to a “constant cold” and, therefore, it is also important to document the frequency and duration of perceived upper respiratory viruses [1].

Before seeking medical attention, patients often attempt using over-the-counter or other medications to manage their symptoms. Assessing patient response to such treatments may provide information that can aid in the diagnosis and subsequent management of AR. For example, symptom improvement with newer, second-generation antihistamines (discussed later) is strongly suggestive of an allergic etiology. However, it is important to note that response to first-generation antihistamines [e.g., diphenhydramine (Benadryl), brompheniramine maleate (Dimetane), chlorpheniramine maleate (Chlor-Tripolon), clemastine (Tavist-1)] does not imply an allergic etiology since the anticholinergic and sedative properties of these agents reduce rhinorrhea and may improve sleep quality regardless of whether the inflammation is allergic. Previous response to intranasal corticosteroids may also be suggestive of an allergic etiology, and likely indicates that such treatment will continue to be beneficial in the future [1]. If an intranasal corticosteroid has previously been used, determining the length of time on treatment, compliance, and administration technique are important for assessing if the treatment trial was appropriate since it can take 2–4 weeks to elicit an adequate response to intranasal steroids.

Important elements of the history for patients with suspected AR are summarized in Table 2.

### Physical examination

The physical examination of patients with suspected AR should include an assessment of outward signs, the nose, ears, sinuses, posterior oropharynx (area of the throat that is at the back of the mouth), chest and skin (see Table 2). Outward signs that may be suggestive of AR include: persistent mouth breathing, rubbing at the nose or an obvious transverse nasal crease, frequent sniffling or throat clearing, and allergic shiners (dark circles under the eyes that are due to nasal congestion). Examination of the nose typically reveals swelling of the nasal mucosa and pale, thin secretions. An internal endoscopic examination of the nose should also be considered to assess for structural abnormalities including septal deviation, nasal ulcerations, and nasal polyps [1].

The ears generally appear normal in patients with AR; however, assessment for Eustachian tube dysfunction using a pneumatic otoscope should be considered. Valsalva’s maneuver (increasing the pressure in the nasal cavity by attempting to blow out the nose while holding it shut) can also be used to assess for fluid behind the ear drum [1].

The sinus examination should include palpation of the sinuses for evidence of tenderness and/or tapping of the maxillary teeth with a tongue depressor for evidence of sensitivity. The posterior oropharynx should also be examined for signs of postnasal drip (mucous accumulation in the back of the nose and throat), and the chest and skin should be examined carefully for signs of concurrent asthma (e.g., wheezing) or dermatitis respectively [1].

### Diagnostic tests

Although a thorough history and physical examination are required to establish the clinical diagnosis of rhinitis, further diagnostic testing is necessary to confirm that underlying allergies cause the rhinitis. Skin-prick testing is considered the primary method for identifying specific allergic triggers of rhinitis. It involves pricking the skin through a drop of commercial extract to introduce the extract into the epidermis. Within 15–20 min, a wheal-and-flare response (an irregular blanched wheal surrounded by an area of redness) greater than the negative control will occur if the test is positive. Testing is typically performed using the allergens relevant to the patient’s environment (e.g., pollen, animal dander, moulds and house dust mites). Antihistamines may interfere with the interpretation of skin tests and, therefore, should be stopped 5–7 days before testing [23].

A reasonable alternative to skin prick testing is the use of allergen-specific IgE tests [e.g., performed by immunosorbent assay—previously performed by radioallergosorbent tests (RASTs)] that provide an in vitro measure of a patient’s specific IgE levels against particular allergens. These in vitro tests can be performed in patients who cannot stop antihistamine therapy to allow for skin testing, those who are unable to come in-person for testing (e.g., during a pandemic or lack of availability of an allergist in their region), or those who also have extensive eczema which would preclude skin testing. Skin prick tests are generally considered to be more sensitive and cost effective than allergen-specific serum IgE tests, and have the further advantage of providing physicians and patients with immediate results. However, allergen-specific serum IgE is an acceptable alternative for guiding diagnosis and treatment [1, 7]. Note that the allergen-specific serum IgE test may not be available to all patients or may be a higher cost to the patient depending on coverage.

Testing patients for food allergies solely based on symptoms of AR is not recommended [16]. Also, sinonasal imaging is not advised in patients with symptoms suggestive of AR alone [24].

## Treatment

**Fig. 2:**
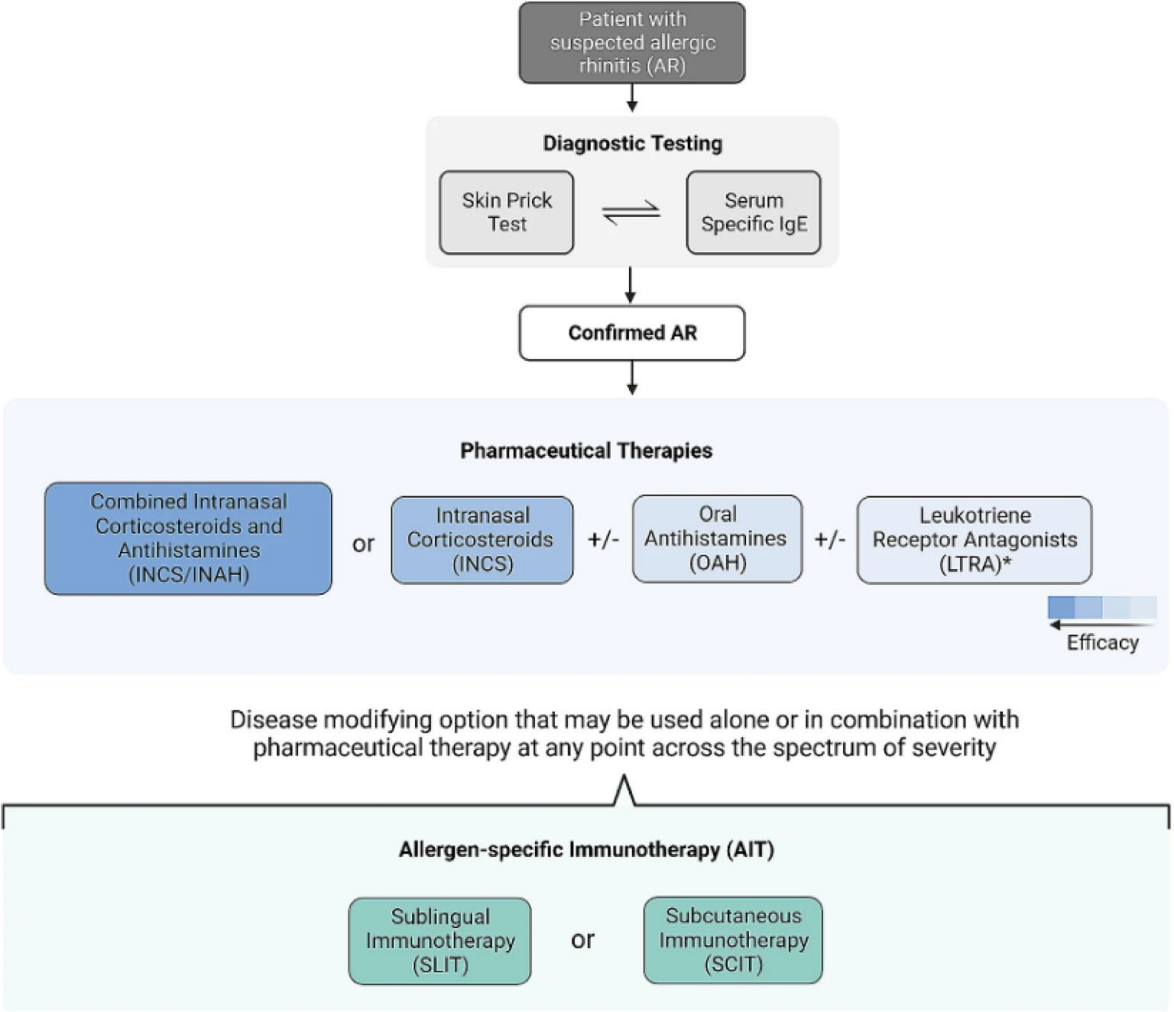
A simplified, stepwise algorithm for the treatment of AR. The choice of therapeutic intervention should be a shared decision-making process involving both the patient and the prescriber, with importance placed on balancing factors such as disease severity, therapy safety, and cost. *IgE* immunoglobulin E (Reproduced from Ellis et al. 2024 [7])

The treatment goal for AR is relief of symptoms. Therapeutic options available to achieve this goal include avoidance measures, nasal saline irrigation, oral antihistamines, intranasal corticosteroids, combination intranasal corticosteroid/antihistamine sprays, leukotriene receptor antagonists (LTRAs), and allergen immunotherapy. Figure 2 provides a simplified, stepwise algorithm for the treatment of AR [7].

Other therapies that may be useful in select patients include decongestants and oral corticosteroids. AR and asthma appear to represent a combined airway inflammatory disease, making treatment of asthma an important consideration in patients with AR. At any point in management, referral to allergist should be considered for confirmatory diagnosis and treatment/management discussions.

### Allergen avoidance

The first-line treatment of AR involves the avoidance of relevant allergens (e.g., house dust mites, moulds, pets, pollens) and irritants (e.g., tobacco smoke) to the extent possible. Patients allergic to house dust mites should be instructed to use allergen-impermeable covers for bedding and to keep the relative humidity in the home below 50% (to inhibit mite growth). However, the humidity level should not be below 30% as this can be irritating to the respiratory mucosa and increase the risk of bacterial infections [25]. Pollen and outdoor mould exposure can be reduced by keeping windows closed, using window screen filters, using an air conditioner, and limiting the amount of time spent outdoors during peak pollen seasons. For patients allergic to animal dander, removal of the animal from the home is recommended and usually results in a significant reduction in symptoms within 4–6 months [1]. However, compliance with this recommendation is poor and, therefore, the use of high-efficiency particulate air (HEPA) filters and restricting the animal from the bedroom or to the outdoors may be needed to attempt to decrease allergen levels. Measures for reducing exposure to mould allergens include cleaning with fungicides, dehumidification to less than 50%, remediation of any water damage, and HEPA filtration. These avoidance strategies can effectively improve the symptoms of AR, and patients should be advised to use a combination of measures for optimal results [1].

### Sterile nasal saline

Nasal irrigation with sterile nasal saline has been shown to improve symptoms and QoL while decreasing the requirement for medication in patients with AR [26, 27]*.* It can also help with nasal moisture and clearing of mucus [28]. Sterile nasal saline is an easy-to-use, low-cost option which patients can prepare themselves, without the potential side effects seen with pharmacotherapy [29]. Due to the risk of transmission of microbial organisms, if tap water is used for nasal irrigation, it should be boiled (1 to 5 min) and then cooled [16, 30, 31].

### Antihistamines

Second-generation oral antihistamines, such as desloratadine (Aerius), fexofenadine (Allegra), loratadine (Claritin), cetirizine (Reactine), bilastine (Blexten) and rupatadine (Rupall), can be used as first-line therapies for patients with AR [7]. Bilastine and rupatadine are available by prescription only (see Table 3 for a list of second-generation antihistamines and their recommended dosing regimens).

**Table 3:**
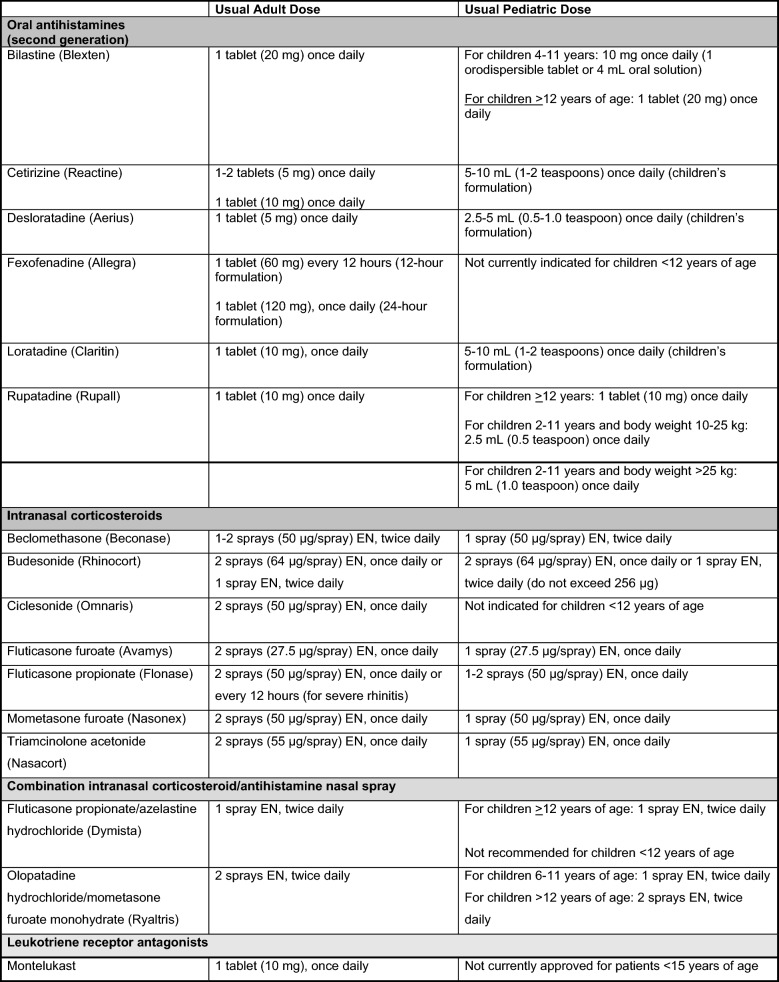
Overview of pharmacologic treatment options for AR

Second-generation oral antihistamines have been found to effectively reduce sneezing, itching and rhinorrhea when taken regularly at the time of maximal symptoms or before exposure to an allergen. While they are less efficacious than intranasal corticosteroids (discussed below), they may be the preferred option for patients based on preference [7, 32].

Although the older (first-generation) sedating antihistamines (e.g., diphenhydramine, chlorpheniramine) are also effective in relieving symptoms, they have been shown to negatively impact cognition and functioning [1]. Because of this, prescribing first-generation antihistamines to patients with AR is not recommended [16, 33].

### Intranasal corticosteroids

Intranasal corticosteroids are a first-line therapy for patients with AR and they can be used alone or in combination with oral antihistamines [7, 16]. When used regularly and correctly, intranasal corticosteroids effectively reduce inflammation of the nasal mucosa and improve mucosal pathology. Studies and meta-analyses have shown that intranasal corticosteroids are superior to antihistamines and LTRAs in controlling the symptoms of AR, including nasal congestion, and rhinorrhea [34–37]. They have also been shown to improve ocular symptoms and reduce lower airway symptoms in patients with concurrent asthma and AR [38–40].

The intranasal corticosteroids approved in Canada are shown in Table 3 and include fluticasone furoate (Avamys), beclomethasone (Beconase), fluticasone propionate (Flonase), triamcinolone acetonide (Nasacort), mometasone furoate (Nasonex), ciclesonide (Omnaris) and budesonide (Rhinocort). Since proper application of the nasal spray is required for optimal clinical response, patients should be counseled on the appropriate use of these intranasal devices. Ideally, intranasal corticosteroids are best started just prior to exposure to relevant allergens and, because their peak effect may take several days to develop, they should be used regularly [8].

The most common side effects of intranasal corticosteroids are nasal irritation and stinging. These side effects can usually be prevented by aiming the spray slightly away from the nasal septum [1]. Evidence suggests that intranasal beclomethasone and triamcinolone, but not other intranasal corticosteroids, may slow growth in children compared to placebo, however, long-term studies examining the impact of usual doses of intranasal beclomethasone on growth are lacking [41–44].

It is important to note that most patients with AR presenting to their primary-care physician have moderate-to-severe symptoms and will require an intranasal corticosteroid. Bousquet et al. noted improved outcomes in patients with moderate-to-severe symptoms treated with a combination of these agents [45].

### Combination intranasal corticosteroid and antihistamine nasal spray

If intranasal corticosteroids are not effective, a combination intranasal corticosteroid/antihistamine spray can be tried, such as combination fluticasone propionate/azelastine hydrochloride (Dymista) or olopatadine hydrochloride/mometasone furoate monohydrate (Ryaltris). Combination intranasal corticosteroid/antihistamine sprays have been shown to be effective within minutes [32], and more effective than the individual components with a safety profile similar to intranasal corticosteroids [46–49]. Combination intranasal corticosteroid/antihistamine sprays are also superior to oral antihistamines used along with intranasal nasal steroids [7]. However, it is unknown whether there are any differences in efficacy between the two approaches after long-term use.

### Leukotriene receptor antagonists (LTRAs)

The LTRAs montelukast and zafirlukast are also effective in the treatment of AR; however, they do not appear to be as effective as intranasal corticosteroids [50–52]. Although one short-term study found the combination of LTRAs and antihistamines to be as effective as intranasal corticosteroids [53], longer-term studies have found intranasal corticosteroids to be more effective than the combination for reducing nighttime and nasal symptoms [35, 54]. Currently montelukast is the only LTRA indicated for the treatment of AR in adults in Canada.

LTRAs should be considered when alternate therapies such as oral antihistamines, intranasal corticosteroids and/or combination corticosteroid/antihistamine sprays are not well tolerated or are insufficient/ineffective in controlling the symptoms of AR [16]. LTRAs have been shown to improve nighttime control of rhinitis symptoms [7]. If a patient has co-morbid asthma, then an LTRA may be of benefit for both conditions [7, 16]. LTRAs confer a risk of serious neuropsychiatric events, particularly in those under 6 years of age [7]. If prescribed, patients should be monitored for these symptoms [16].

### Allergen immunotherapy

Allergen immunotherapy, which involves the subcutaneous or sublingual administration of the patient’s relevant allergens, is an option for patients who do not achieve symptom control with pharmacotherapy or who prefer this form of therapy [7] (see *Allergen Immunotherapy* article in this supplement). It can be used as an alternative or add-on to the pharmacological therapies described above, and it is particularly effective in patients with intermittent (seasonal) AR caused by pollens, including tree, grass and ragweed pollens [55–58]. Allergen immunotherapy has also been shown to be effective for the treatment of AR caused by house dust mites, Alternaria, cockroach, and cat and dog dander (although it should be noted that therapeutic doses of dog allergen are difficult to attain with the allergen extracts available in Canada). The effect of allergen immunotherapy varies depending on the allergen administered.

In subcutaneous allergen immunotherapy (SCIT), gradually increasing quantities of the patient’s relevant allergens are injected over time until a dose is reached that is effective in inducing immunologic tolerance to the allergen. Typically, SCIT is given on a perennial basis with weekly incremental increases in dose over the course of 6–8 months, followed by maintenance injections of the maximum tolerated dose every 3 to 4 weeks for 3 to 5 years. After this period, many patients experience a prolonged, protective effect and, therefore, consideration can be given to stopping therapy. Pre-seasonal subcutaneous preparations that are administered for 4–12 weeks on an annual basis are also available but are currently difficult to access in Canada [57].

Sublingual immunotherapy (SLIT) is an alternative way of desensitizing patients and involves placing a tablet of allergen extract under the tongue for 1 to 2 min until it is dissolved. It is currently available for the treatment of tree, grass, ragweed and dust mite allergy. At present, five sublingual tablet immunotherapy products are available in Canada: Oralair®, Grastek® (both targeting grass pollen), Ragwitek® (ragweed) Itulatek® (birch tree), and Acarizax™ (dust mites) [59–63]. SLIT should only be administered using these Health Canada-approved products.

SLIT offers multiple potential benefits over the subcutaneous route including the comfort of avoiding injections, the convenience of home administration, and a favourable safety profile. The most common side effects of SLIT are local reactions such as oral pruritus, throat irritation, and ear pruritus [57]. These symptoms typically resolve after the first week of therapy. There is a very small risk of more severe systemic allergic reactions with this type of immunotherapy and, therefore, some allergists may offer the patient an epinephrine auto-injector in case a reaction occurs at home. However, the risk of systemic allergic reactions is much lower with SLIT compared to traditional injections (SCIT) [57].

Both SCIT and SLIT are effective treatment options, but there is insufficient data to determine the benefit of one over the other. Shared decision making with the patient that takes into account factors such as cost, risk and practical considerations can help guide which is better suited for an individual patient [7]. Since both forms of immunotherapy carry a risk of anaphylactic reactions, they should only be prescribed by physicians who are adequately trained in the treatment of allergy and who are equipped to manage possible life-threatening anaphylaxis [1].

Evidence suggests that at least 3 years of allergen immunotherapy (SCIT or SLIT) provides beneficial effects in patients with AR that can persist for several years after discontinuation of therapy [64–70]. In Canada, most allergists consider stopping immunotherapy after 5 years of adequate treatment. Recent data has made it clear that only 2 years of immunotherapy, either via the subcutaneous or sublingual route, is not sufficient to provide long-lasting effects [69, 71].

Both forms of immunotherapy are contraindicated in patients with severe, unstable or uncontrolled asthma, and should ideally be avoided in patients on beta-blocker therapy. SLIT is contraindicated in those with active oral inflammation or sores or those with eosinophilic esophagitis [57].

### Other therapeutic options

Oral and intranasal decongestants (e.g., pseudoephedrine, phenylephrine) are useful for relieving nasal congestion in patients with AR. However, the side-effect profile associated with oral decongestants (i.e., agitation, insomnia, headache, palpitations) may limit their long-term use. Furthermore, these agents are contraindicated in patients with uncontrolled hypertension and severe coronary artery disease. Prolonged use of intranasal decongestants carries the risk of rhinitis medicamentosa (rebound nasal congestion) [72] and, therefore, use should be limited to the short term [16]. In patients who do not experience benefits with intranasal corticosteroids alone or a combination intranasal corticosteroid/antihistamine, intranasal decongestants in combination with intranasal corticosteroids for up to 4 weeks (but ideally no longer than a few days) may be considered [16].

Oral corticosteroids have also been shown to be effective in patients with severe AR that is refractory to treatment with oral antihistamines and intranasal corticosteroids [1, 8]. Therefore, a short course (5–7 days) of oral corticosteroids may be considered in these patients [16].

Although not as effective as intranasal corticosteroids, intranasal sodium cromoglycate (Cromolyn) has been shown to reduce sneezing, rhinorrhea and nasal itching and, therefore, it is a reasonable therapeutic option for some patients [1]. The anti-IgE antibody, omalizumab, has also been shown to be effective in seasonal AR [1], however, it is not currently approved for the treatment of AR.

Surgical therapy may be helpful for select patients with rhinitis, polyposis, or chronic sinus disease that is refractory to medical treatment [1].

### Complementary and alternative medicines (CAM)

Given the popularity of complementary and alternative medicines (CAM) in the general population, it is reasonable for physicians to ask patients about their use of CAM in a nonjudgmental manner. Given the limited number of well-designed clinical trials examining the efficacy of CAM in AR, it is difficult for clinicians to evaluate these therapies and provide guidance.

Various CAM have been used for the management of AR, including traditional Chinese medicines, acupuncture, homeopathy, and herbal therapies [73]. At present, there is insufficient evidence to support the use of CAM for the management of AR [16].

### Special considerations: pregnancy

It is important to note that AR may worsen during pregnancy and, as a result, may necessitate pharmacologic treatment. The benefit-to-risk ratio of pharmacological agents for AR needs to be considered before recommending any medical therapy to pregnant women. Sterile nasal saline is a first-line option as it is safe and effective in pregnant women [16]. Intranasal sodium cromoglycate can be used for AR in pregnancy since no teratogenic effects have been noted with the cromones in humans or animals. With the exception of triamcinolone, most intranasal corticosteroids are safe in pregnancy, with newer data supporting the safety of mometasone and fluticasone [16]. Antihistamines may also be considered for AR in pregnancy, specifically cetirizine and loratadine. Oral decongestants should not be used in pregnancy, especially in the first trimester [16]. Starting or increasing allergen immunotherapy during pregnancy is not recommended because of the risk of anaphylaxis for the mother and potential complications to the fetus. However, maintenance doses are considered to be safe and effective during pregnancy [1].

## Conclusions

AR is a common disorder that can significantly impact patient QoL. The diagnosis is made through a comprehensive history and physical examination. Further diagnostic testing using skin-prick tests or allergen-specific IgE tests are helpful to confirm that underlying allergen sensitization is the cause of rhinitis. Many therapeutic options are available for the treatment of AR that are effective and well tolerated, including intranasal corticosteroids, second-generation oral antihistamines and allergen immunotherapy.

## Data Availability

Data sharing not applicable to this article as no datasets were generated or analyzed during the development of this review.

## Abbreviations

ACE: Angiotensin-converting enzyme
AR: Allergic rhinitis
ARIA: Allergic Rhinitis and its Impact on Asthma
ASA: Acetylsalicylic acid
CAM: Complementary and alternative medicines
HEPA: High-efficiency particulate air
IgE: Immunoglobulin E
IL: Interleukin
LAR: Local allergic rhinitis
NSAIDs: Non-steroidal anti-inflammatory drugs
LTRAs: Leukotriene receptor antagonists
QoL: Quality of life
Th2: T helper 2
